# 907. Age-stratified seroprevalence of respiratory syncytial virus in Korean population

**DOI:** 10.1093/ofid/ofad500.952

**Published:** 2023-11-27

**Authors:** Eliel Nham, A Yeung Jang, Hakjun Hyun, Jin Gu Yoon, Ji Yun Noh, Hee Jin Cheong, Woo Joo Kim, Hyun Jung Ji, Ho Seong Seo, Joon Young Song

**Affiliations:** Division of Infectious Diseases, Department of Internal Medicine, Korea University College of Medicine, Seoul, South Korea, Seoul, Seoul-t'ukpyolsi, Republic of Korea; Korea University Guro Hospital, Seoul, Seoul-t'ukpyolsi, Republic of Korea; Korea University Guro Hospital, Seoul, Seoul-t'ukpyolsi, Republic of Korea; Division of Infectious Diseases, Department of Internal Medicine, Korea University College of Medicine, Seoul, South Korea, Seoul, Seoul-t'ukpyolsi, Republic of Korea; Division of Infectious Diseases, Department of Internal Medicine, Korea University College of Medicine, Seoul, South Korea, Seoul, Seoul-t'ukpyolsi, Republic of Korea; Division of Infectious Diseases, Department of Internal Medicine, Korea University College of Medicine, Seoul, South Korea, Seoul, Seoul-t'ukpyolsi, Republic of Korea; Division of Infectious Diseases, Department of Internal Medicine, Korea University College of Medicine, Seoul, South Korea, Seoul, Seoul-t'ukpyolsi, Republic of Korea; Korea Atomic Energy Research Institute, Seoul, Seoul-t'ukpyolsi, Republic of Korea; Korea Atomic Energy Research Institute, Seoul, Seoul-t'ukpyolsi, Republic of Korea; Division of Infectious Diseases, Department of Internal Medicine, Korea University College of Medicine, Seoul, South Korea, Seoul, Seoul-t'ukpyolsi, Republic of Korea

## Abstract

**Background:**

Respiratory syncytial virus the two modified prefusion (preF) protein antigens (DS-Cav1 and SC-TM) and the fusion (G) protein, (RSV), a major childhood respiratory pathogen, is gaining attention as an important pathogen in adults also, especially in older people and the immunocompromised. Studies on RSV seroprevalence in the adult population are limited, even though it is important to know the level of preexisting immunity to RSV now that approval of RSV vaccines seems imminent. Therefore, we aimed to analyze antibody levels against RSV across age groups.

*This study was funded by the grant of the Ministry of Food and Drug Safety of South Korea (21172MFDS179).

**Methods:**

A total of 150 serum samples were collected from 30 subjects of each age group (under 5 years, 5–18 years, 19–49 years, 50–64 years, and ≥65 years). Considering higher RSV disease burden in infants than older children, children under 5 were further divided into two age groups including 0–12 months and 13–59 months. Age-stratified seroprevalence was estimated using ELISA targeting the two modified prefusion (preF) protein antigens (DS-Cav1 and SC-TM) and the fusion (G) protein, the major immunogens and popular vaccine targets. Seropositivity was determined based on the concentration of medium-titer RSV antiserum manufactured by the United States National Institute of Health.

**Results:**

Antibody titers against SC-TM were similar across age groups. Antibody titers against DS-Cav1 were higher than those against SC-TM in all age groups except children aged 0–12 months. There was no significant difference in antibody titers between children aged 0–12 months and 13–59 months. Only about one fifth participants aged under 5 and over 65 were seropositive to RSV preF.

**Figure:**
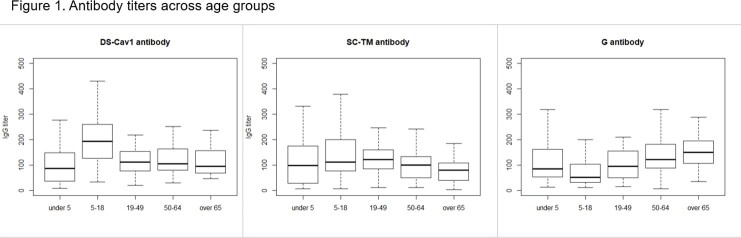

**Conclusion:**

RSV seroprevalence was low overall. This study supports the importance of vaccination against RSV in infants and people over 65.

**Disclosures:**

**Hee Jin Cheong, M.D.,Ph.D.**, Sequiris: Advisor/Consultant

